# Supplementary material for: Docetaxel Administration via Novel Hierarchical Nanoparticle Reduces Proinflammatory Cytokine Levels in Prostate Cancer Cells
Source: Cancers (Basel). 2025 May 23;17(11):1758. doi: 10.3390/cancers17111758 (PMC12153775; doi:10.3390/cancers17111758)
Supplement: Supplementary file 1 [file cancers-17-01758-s001.zip › cancers-3531468-supplementary.pdf]

**Supplemental Material: Docetaxel Administration *via* Novel Hierarchical Nanoparticle Reduces Proinflammatory Cytokine Levels in Prostate Cancer Cells.**

**Ravikumar Aalinkeel, Satish Sharma, Supriya Mahajan, Paras Prasad and Stanley A. Schwartz.**

# (A)

| Number | Group | File Name | Date | Dilution factor | Total concentration |
|---|---|---|---|---|---|
| 1 | HNP-Doc | LNCap | 20250424 | 2 | 6950617 |
| 2 | HNP-Doc | LNCap | 20250424 | 2 | 6881711 |
| 3 | HNP-Doc | LNCap | 20250424 | 2 | 6909560.5 |
| 4 | HNP-Doc | LNCap | 20250424 | 2 | 6954547 |
| 5 | HNP-Doc | LNCap | 20250424 | 2 | 7068618.5 |
| 6 | HNP-Doc | LNCap | 20250424 | 2 | 6856158 |
| 7 | HNP-Doc | LNCap-Doc/R | 20250424 | 2 | 8125179 |
| 8 | HNP-Doc | LNCap-Doc/R | 20250424 | 2 | 8481194 |
| 9 | HNP-Doc | LNCap-Doc/R | 20250424 | 2 | 8136663.5 |
| 10 | HNP-Doc | LNCap-Doc/R | 20250424 | 2 | 6936548.5 |
| 11 | HNP-Doc | LNCap-Doc/R | 20250424 | 2 | 8222796 |
| 12 | HNP-Doc | LNCap-Doc/R | 20250424 | 2 | 964685.563 |
| 13 | Doc- Transfected | LNCap | 20250424 | 2 | 4358311.5 |
| 14 | Doc- Transfected | LNCap | 20250424 | 2 | 7189204.5 |
| 15 | Doc- Transfected | LNCap | 20250424 | 2 | 6436979.5 |
| 16 | Doc- Transfected | LNCap | 20250424 | 2 | 7401665 |
| 17 | Doc- Transfected | LNCap | 20250424 | 2 | 7114556 |
| 18 | Doc- Transfected | LNCap | 20250424 | 2 | 6247487.5 |
| 19 | Doc- Transfected | LNCap-Doc/R | 20250424 | 2 | 3560149 |
| 20 | Doc- Transfected | LNCap-Doc/R | 20250424 | 2 | 6971001.5 |
| 21 | Doc- Transfected | LNCap-Doc/R | 20250424 | 2 | 6959517.5 |
| 22 | Doc- Transfected | LNCap-Doc/R | 20250424 | 2 | 6126902 |
| 23 | Doc- Transfected | LNCap-Doc/R | 20250424 | 2 | 3588860 |
| 24 | Doc- Transfected | LNCap-Doc/R | 20250424 | 2 | 3537180.5 |
| 25 | Control | LNCap | 20250424 | 2 | 2595463.5 |
| 26 | Control | LNCap | 20250424 | 2 | 2227964.25 |
| 27 | Control | LNCap | 20250424 | 2 | 2744760.25 |
| 28 | Control | LNCap | 20250424 | 2 | 2624174.5 |
| 29 | Control | LNCap | 20250424 | 2 | 2480620 |
| 30 | Control | LNCap | 20250424 | 2 | 2497846.5 |
| 31 | Control | LNCap-Doc/R | 20250424 | 2 | 4036749.75 |
| 32 | Control | LNCap-Doc/R | 20250424 | 2 | 1498707.875 |
| 33 | Control | LNCap-Doc/R | 20250424 | 2 | 5173700.5 |
| 34 | Control | LNCap-Doc/R | 20250424 | 2 | 3847258 |
| 35 | Control | LNCap-Doc/R | 20250424 | 2 | 4570772 |
| 36 | Control | LNCap-Doc/R | 20250424 | 2 | 2727533.5 |

| Live concentrat | Dead concentrat | Viability (%) | % Apoptosis | Avg % Apoptosis | Avg Size |
|---|---|---|---|---|---|
| 4295481.306 | 2655135.694 | 62% | 38% | | 11.696 |
| 4369886.485 | 2511824.515 | 64% | 37% | | 11.715 |
| 4366842.24 | 2542718.26 | 63% | 37% | | 11.575 |
| 4270091.86 | 2684455.14 | 61% | 39% | | 11.581 |
| 4552190.31 | 2516428.19 | 64% | 36% | | 11.344 |
| 4353660.33 | 2502.497.67 | 64% | 37% | **37%** | 11.422 |
| 4652331.984 | 3472847.016 | 57% | 42% | | 10.665 |
| 4675854 | 3805340 | 55% | 44% | | 12.028 |
| 4089036.125 | 4047627.375 | 50% | 49% | | 11.651 |
| 3794292.029 | 3142256.471 | 55% | 45% | | 12.242 |
| 4168705 | 4054091 | 50% | 49% | | 11.895 |
| 511283.3484 | 453402.2146 | 52% | 47% | **46%** | 11.616 |
| 3068251.296 | 1290060.204 | 70% | 30% | | 12.738 |
| 5420614 | 1768590.25 | 75% | 25% | | 11.147 |
| 4886592 | 1550387.5 | 76% | 24% | | 11.239 |
| 5782371 | 1619293.625 | 78% | 22% | | 11.076 |
| 5079792.984 | 2034763.016 | 74% | 26% | | 11.325 |
| 4460706.075 | 1786781.425 | 71% | 29% | **26%** | 11.118 |
| 2,944,243.22 | 615,905.78 | 83% | 17% | | 12.367 |
| 5604685.206 | 1345403.29 | 81% | 19% | | 11.33 |
| 5621590.5 | 1337927 | 81% | 19% | | 11.364 |
| 5030146 | 1096755.625 | 82% | 18% | | 11.426 |
| 2939994 | 648865.875 | 82% | 18% | | 12.303 |
| 2945736.25 | 591444.125 | 83% | 17% | **18%** | 11.513 |
| 2535767.84 | 59695.6605 | 98% | 2% | | 14.885 |
| 2090152.125 | 26735.571 | 99% | 1% | | 14.134 |
| 2629916.5 | 41171.4 | 99% | 2% | | 15.166 |
| 2,590,060.23 | 34,114.27 | 99% | 1% | | 14.299 |
| 2,448,371.94 | 32,248.06 | 99% | 1% | | 14.347 |
| 2,440,396.03 | 57,450.47 | 99% | 1% | **1%** | 14.323 |
| 3943905 | 92845.24 | 98% | 2% | | 12.903 |
| 1462739 | 35968.98 | 98% | 2% | | 12.155 |
| 5101269 | 72431.28 | 99% | 1% | | 12.833 |
| 3785701.87 | 61556.128 | 98% | 2% | | 13.785 |
| 4488498.1 | 82273.896 | 98% | 2% | | 13.821 |
| 2678437.897 | 49095.63 | 98% | 2% | **2%** | 12.87 |

**(B)**

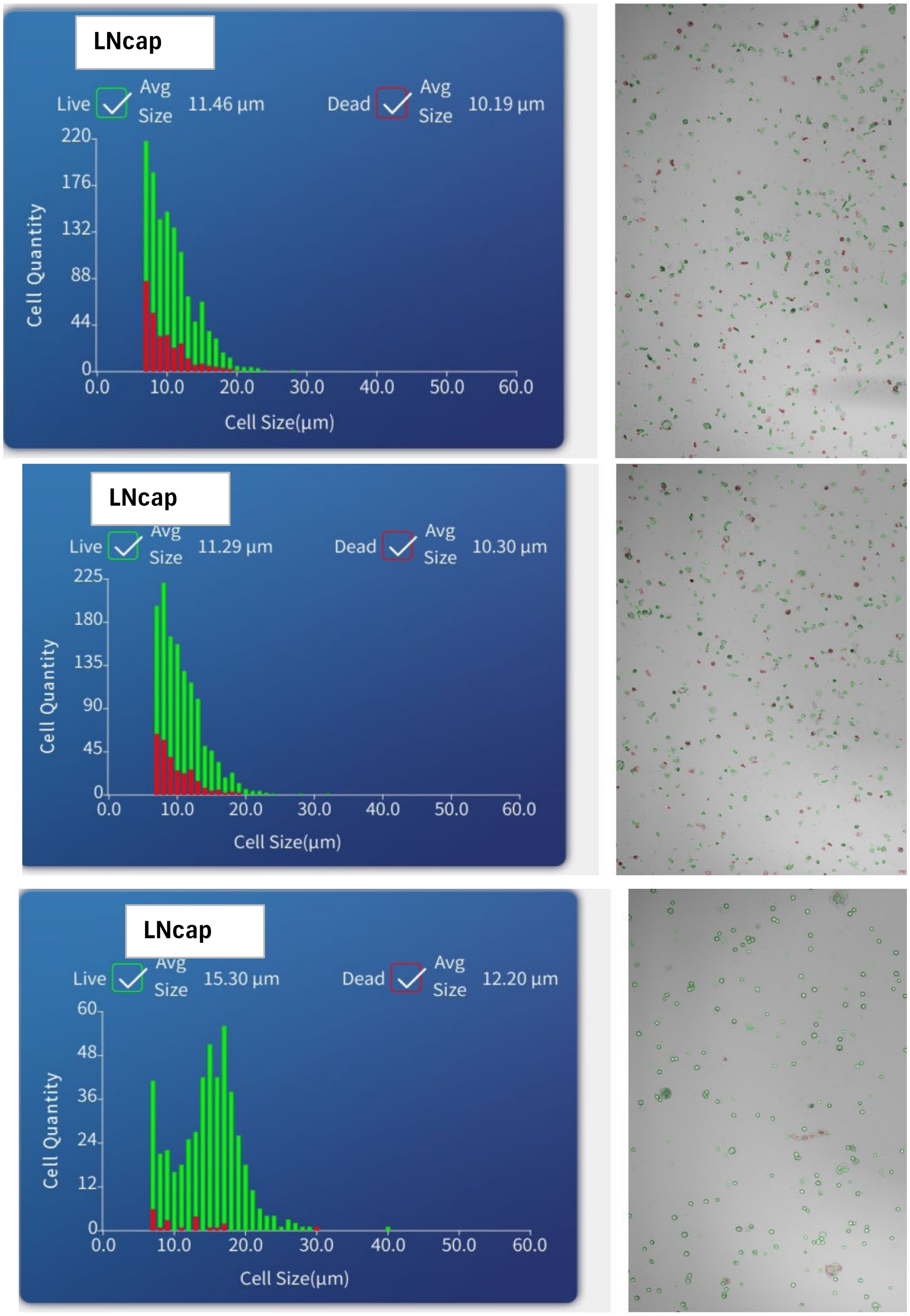
LNcap
Live Avg Size 11.46 µm
Dead Avg Size 10.19 µm
Cell Quantity
220
176
132
88
44
0
0.0 10.0 20.0 30.0 40.0 50.0 60.0
Cell Size(µm)
LNcap
Live Avg Size 11.29 µm
Dead Avg Size 10.30 µm
Cell Quantity
225
180
135
90
45
0
0.0 10.0 20.0 30.0 40.0 50.0 60.0
Cell Size(µm)
LNcap
Live Avg Size 15.30 µm
Dead Avg Size 12.20 µm
Cell Quantity
60
48
36
24
12
0
0.0 10.0 20.0 30.0 40.0 50.0 60.0
Cell Size(µm)

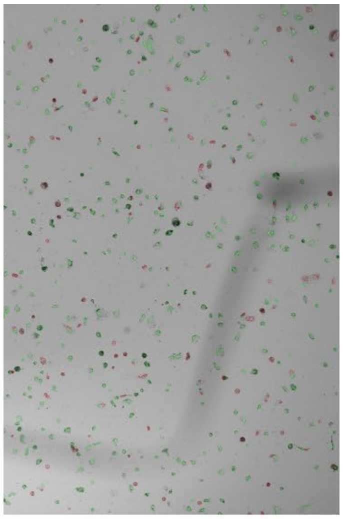
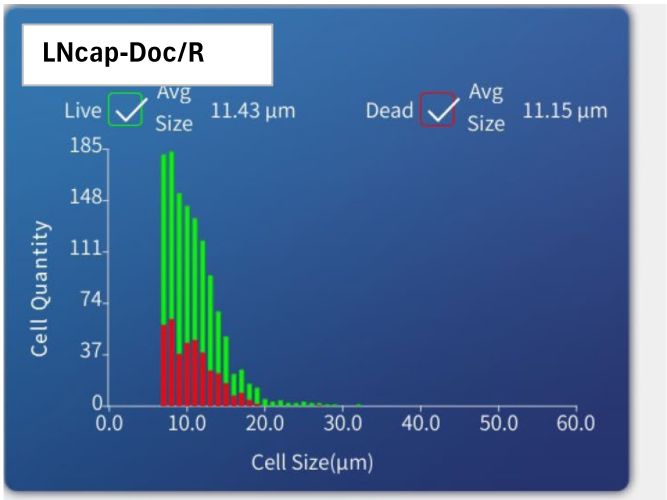
LNcap-Doc/R
Live Avg Size 11.43 µm
Dead Avg Size 11.15 µm
185
148
111
74
37
0
Cell Quantity
0.0 10.0 20.0 30.0 40.0 50.0 60.0
Cell Size(µm)
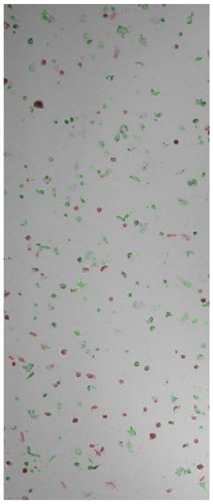
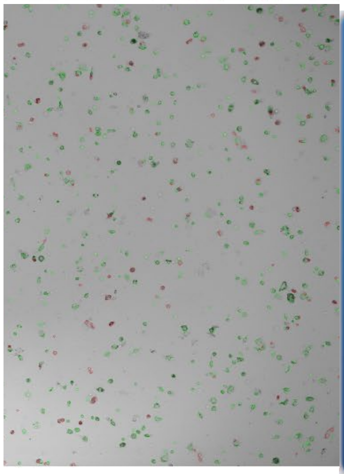
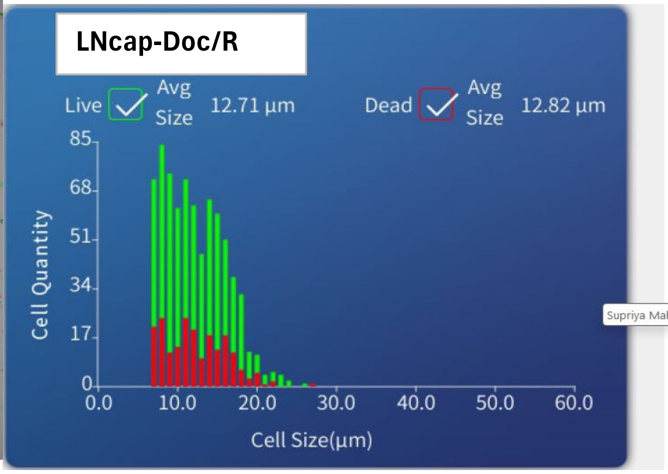
LNcap-Doc/R
Live Avg Size 12.71 µm
Dead Avg Size 12.82 µm
85
68
51
34
17
0
Cell Quantity
0.0 10.0 20.0 30.0 40.0 50.0 60.0
Cell Size(µm)
Supriya Mahajan (smahajan@buffalo.edu) is signed in
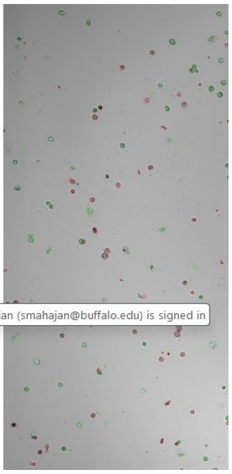
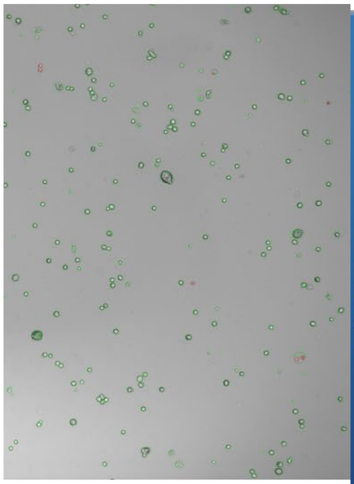
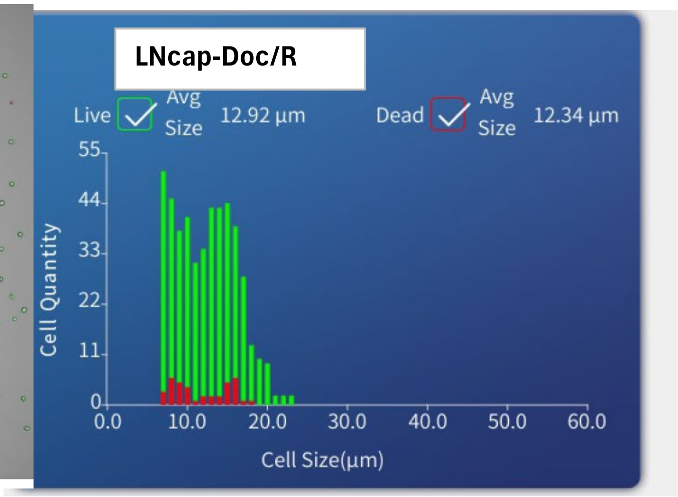
LNcap-Doc/R
Live Avg Size 12.92 µm
Dead Avg Size 12.34 µm
55
44
33
22
11
0
Cell Quantity
0.0 10.0 20.0 30.0 40.0 50.0 60.0
Cell Size(µm)
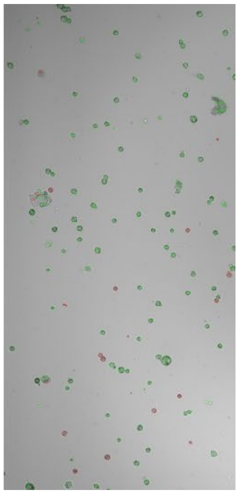

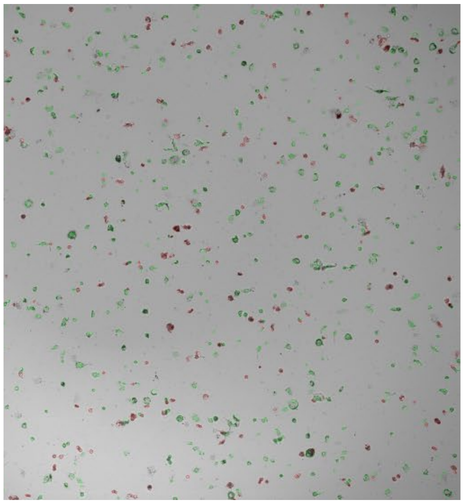

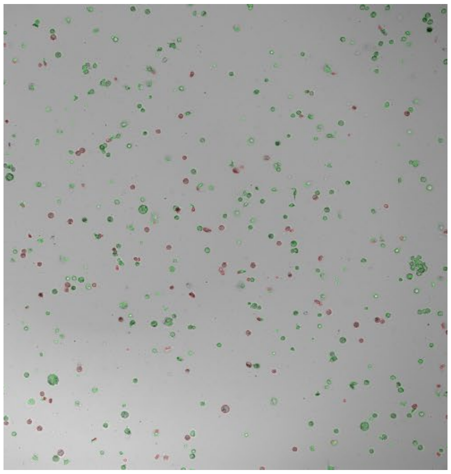

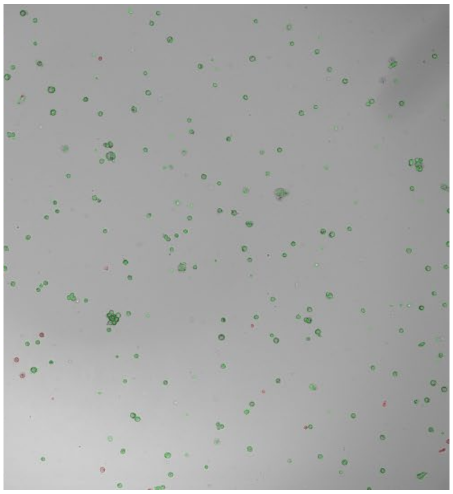

**Figure S1.** Original data generated by RWD-C100-Pro automated cell counter 100 of Annexin-V/PI stained LNCaP and LNCaP-Doc/R . The automated cell counter can identify cell counts, viability, diameter and fluorescent counts of each Fluorophore with just one click from which rate of Apoptosis is calculated. Fluorescence cube, C100 can display images in BF and FL channels simultaneously and present cell counts with different fluorophores. The cell count data is utilized to calculate % dead and viable cells and rate of apoptosis is calculated based on the ratio dead to viable cells. Insert A is the original data generated by the machine and (B) A representative image of the fluorophore(Green and Red) stained LNCaP and LNCaP/Doc-R cells.
